# Socio-environmental modeling shows physics-like confidence with water modeling surpassing it in numerical claims

**DOI:** 10.1016/j.isci.2025.112184

**Published:** 2025-03-13

**Authors:** Arnald Puy, Ethan Bacon, Alba Carmona, Samuel Flinders, David Gefen, Mohammad Khanjani, Kai R. Larsen, Alessio Lachi, Seth N. Linga, Samuele Lo Piano, Lieke A. Melsen, Emily Murray, Razi Sheikholeslami, Ariana Sobhani, Nanxin Wei, Andrea Saltelli

**Affiliations:** 1 School of Geography, Earth and Environmental Sciences, University of Birmingham, Birmingham B15 2TT, UK; 2 Department of Modern Languages, College of Arts and Law, University of Birmingham, Birmingham B15 2TT, UK; 3 School of Languages, Cultures and Societies, Faculty of Arts, Humanities and Cultures, University of Leeds, Leeds LS2 9JT, UK; 4 LeBow College of Business, Drexel University, Philadelphia, PA 19104, USA; 5 Department of Civil Engineering, Sharif University of Technology, Azadi Avenue, Tehran 11155-4313, Iran; 6 Organizational Leadership and Information Analytics, Leeds School of Business, University of Colorado Boulder, Boulder, CO, USA; 7 Saint Camillus International University of Health and Medical Sciences (UniCamillus), Via Sant’Alessandro 8, 00131 Rome, Italy; 8 University of Reading, School of the Built Environment, JJ Thompson Building, Whiteknights Campus, Reading RG6 6AF, UK; 9 Hydrology and Environmental Hydraulics Group, Wageningen University, P.O. Box 9101, 6700 HB Wageningen, the Netherlands; 10 School of Biosciences, University of Birmingham, Birmingham B15 2TT, UK; 11 Barcelona School of Management, Pompeu Fabra University, Carrer de Balmes 132, 08008 Barcelona, Spain; 12 Centre for the Study of the Sciences and the Humanities, University of Bergen, Parkveien 9, PB 7805, 5020 Bergen, Norway

**Keywords:** Computational mathematics, Environmental science, Interdisciplinary application studies, Water resources engineering

## Abstract

Several modern scientific fields rely on computationally intensive mathematical models to study uncertain, complex socio-environmental phenomena such as the spread of a virus, climate change, or the water cycle. However, the degree of epistemic commitment of these fields is unclear. By using machine learning to extract the knowledge claims of around 755,000 abstracts from 14 scientific fields spanning the human and physical sciences, we show that epidemic, integrated assessment, and water modeling display a degree of linguistic assertiveness akin to physics. Water modeling surpasses even the most accurate physical sciences in substantiating knowledge claims with numbers, which are largely produced without accompanying uncertainty and sensitivity analysis. By exploring the balance between doubt and certainty in academic writing, our study reflects on whether the strong conviction and quantification of fields modeling socio-environmental processes, especially water modeling, are epistemically justified.

## Highlights

• IAMs, epidemic and water modelling simulate complex socio-environmental systems
• Their assertivity in knowledge claims is similar to that in the physical sciences
• Water modelling surpasses even thermodynamics in numerical claims
• We reflect on whether this strong assertivity and numerification is warranted

## Graphical abstract

**Figure:**
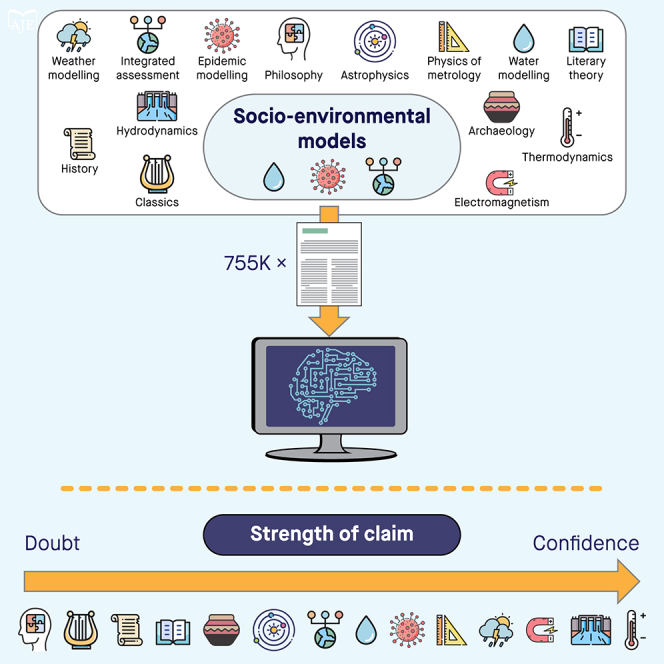

## Introduction

Many scientific fields rely heavily on computer simulations to produce insights into complex, cross-disciplinary phenomena. Examples are epidemic, integrated assessment, and water modeling, which formalize biological, human, environmental, and physical interactions within mathematical frameworks to study the spread of diseases, climate change, or the water cycle. These fields also have strong policy implications: epidemic models were used to guide actions against the COVID-19 pandemic,^1^ integrated assessment models (IAMs) are employed to explore sustainable energetic and climatic pathways,^2^ and water models are leveraged to inform the water-related sustainable development goals.^3^^,^^4^

Because their simulations do not often correspond to closed real-world systems that can be manipulated in the lab, the ability of models to generate knowledge is uncertain. Unlike experiments, which can study the system’s behavior to reveal the effects of unknown properties without requiring detailed prior knowledge of these unknowns, models must explicitly model (or omit) unknown factors.^5^ This feature makes fields that strongly rely on models more epistemically vulnerable to unknowns than fields that can manipulate the system of interest—a problem that is exacerbated in fields modeling environmental and/or social systems, where the open-ended nature and complexity of variables make understanding relationships particularly challenging. The intrinsic uncertainty of modeling has led some academics to view it as a craft, more like the humanities than the “hard” sciences, due to the considerable freedom in framing, executing, and interpreting model-based research.^6^^,^^7^

Given the ambiguous correspondence between models and their real-world counterparts, a crucial question arises: to what extent are fields modeling socio-environmental systems committed to their knowledge claims? Specifically, how do they strike a balance between certainty and doubt when presenting model-based insights? In academic writing, clear, compelling propositions should be supported by solid evidence, while nuance and modulation should characterize assertions with a limited empirical base, uncertain, or of an exploratory nature.^8^^,^^9^^,^^10^ Although rhetoric strategies may be influenced by extra-scientific factors (e.g., the desire to persuade the reader, competition for funds, fame, and recognition), the intensity of fuzzy and assertive statements should ideally delineate the boundary between assumptions and facts and match each scientific field’s capacity to map onto the object of study. In other words, scientific communication should align with the empirical base of the phenomena studied to maintain public trust in scientific findings.

Here we examine the strength of knowledge claims in integrated assessment, epidemic, and water modeling through the hierarchy of sciences (HoS) framework.^11^^,^^12^^,^^13^ Formulated by Comte 200 years ago,^14^ HoS postulates that moving from the human/social sciences to the physical sciences (that is, from more complex and particular to simple and general phenomena), there is an increase in consensus and in the ability to acquire scientific knowledge. This is because our capacity to understand the object of study increases when there are less elements involved, less interactions and non-linear effects, and more opportunities to isolate the process of interest.^12^^,^^13^ Since its inception, the HoS hypothesis has been widely debated, receiving both empirical support (e.g., from studies ranking physics above sociology based on scientific consensus indicators^15^) and criticism (e.g., from studies arguing that consensus varies more within disciplines—between established theories and emerging research—than across them^11^).

We adopt the HoS framework based on the connection it establishes between academic rhetoric and epistemic strength: according to the HoS narrative, human sciences may favor tentative claims given the difficulties involved in accurately mapping high-dimensional, often abstract, generally uncertain phenomena (best studied through reflection and approximation). Physics, on the other side, may legitimate stronger claims given their focus on predictable, fundamental regularities (and hence appraisable through empirical research, experimentation, isolation, and quantification). The human and the physical sciences can therefore be understood as the endpoints of an epistemic continuum, from less to more assertive knowledge claims, against which the degree of epistemic commitment in computationally intensive, cross-disciplinary fields can be evaluated.

By using machine learning to extract knowledge claims from abstracts and assessing their degree of assertiveness and use of numbers, we find that knowledge claims become increasingly assertive and definitive as one moves from the humanities to the physical sciences. Fields that model complex, cross-disciplinary phenomena exhibit a level of conviction comparable to that of physics. Notably, integrated assessment and water modeling support their claims with more numerical detail than even the most exact physical disciplines, including thermodynamics. This tendency is commendable only if one assumes that these fields can achieve a level of accuracy comparable to the most precise physical sciences in quantifying real-world phenomena. We challenge this assumption in the context of water modeling by examining sensitivity analysis (SA) practices through a close-reading of 978 documents.

## Results

### Socio-environmental modeling fields show physics-like assertiveness

We retrieve papers from 14 scientific fields, including the human (philosophy, classics, history, literary theory, and archeology) and the physical sciences (astrophysics, metrology, electromagnetism, hydrodynamics, and thermodynamics). For socio-environmental modeling we select epidemic, integrated assessment, and water modeling due to their scientific and policy relevance and their simulation of both natural (e.g., carbon, hydrological processes, and environmental transmission factors) and human-driven (e.g., water management, energy consumption, and public health interventions) phenomena. We also select weather modeling as a reference point to place these modeling fields in the HoS given its physics-based nature, simulation of complex phenomena, and proved predictive success (Method details).

Since our focus is on the degree of epistemic commitment, we use SciBERT^16^ and machine learning to extract from the ∼755K abstracts making our corpus only those sentences producing knowledge claims; that is, presenting research results. We then tally the number of boosting and hedging terms per sentence, for a total of circa 5M sentences and 372 boosters and hedgers (Tables S1 and S2). Boosters are terms used to emphasize a strong commitment to a proposition and implicitly exclude alternative interpretations (e.g., “affirm,” “assert,” and “compelling”). Hedgers nuance statements and indicate doubt and modesty or acknowledge the existence of different perspectives (e.g., “suggest,” “believe,” and “speculate”)^8^^,^^9^ (Method details). Boosters and hedgers are commonly used as cues to assess the strength of claims in scientific writing.^9^^,^^17^^,^^18^

**Figure 1:**
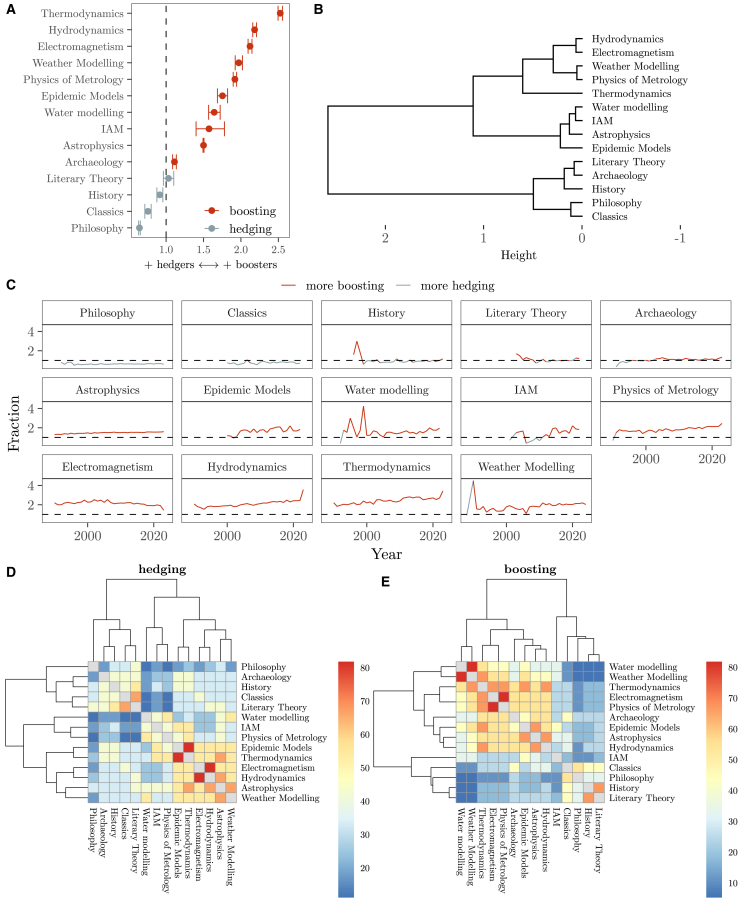
Boosters and hedgers in knowledge claims (A) Booster-to-hedger ratio. The error bars show the 95% confidence intervals after bootstrapping the booster-to-hedger ratio $10^{3}$ times at the field level (percentile method). The vertical, dashed line shows equal number of boosters and hedgers. IAM stands for integrated assessment modeling. (B) Dendrogram after a hierarchical clustering showing the similarities across scientific fields based on the mean bootstrapped booster-to-hedger ratio (number of replicas = $10^{3}$). (C) Evolution of the booster-to-hedger ratio in knowledge claims over time. The horizontal, dashed line shows equal number of boosters and hedgers. (D and E) Heatmaps illustrating the percentage of shared hedgers and boosters across fields (only the ten most frequent terms are taken into account).

The results indicate the existence of a continuum, with the human sciences favoring nuance in their epistemic commitments and the physical sciences preferring confidence (Figure 1A). Philosophy, classics, and history lean toward hedgers; literary theory balances hedgers; and boosters and archeology slightly leans toward more boosters, probably due to its blending of a historical perspective with a more empirical, natural sciences approach.^19^^,^^20^ All the physical fields selected favor more boosters than hedgers: in ascending order of assertiveness we find astrophysics, metrology, electromagnetism, hydrodynamics, and thermodynamics. The same applies to all socio-environmental modeling fields considered, where a preference for boosters over hedgers positions them above astrophysics in terms of linguistic certainty. When a hierarchical clustering is conducted to explore the underlying structure of the data, the three epistemic traditions are distinguished (Figure 1B): classics, philosophy, archeology, history, and literary theory are grouped together, as are thermodynamics, electromagnetism, hydrodynamics, weather modeling, and metrology. The socio-environmental modeling fields are also grouped together and connected to the physical sciences, with epidemic, integrated assessment, and water modeling being closer to astrophysics.

Over the past thirty years, the balance of certainty and doubt in the selected human fields has remained stable. In contrast, the physical and the socio-environmental modeling fields have increasingly favored assertive statements (Figure 1C). This may suggest that the strength of epistemic propositions in the human sciences is not as influenced by transient cultural, sociological, or technological practices than in the other two disciplines. Notably, two of the top four fields with the largest growth in assertiveness fall into the socio-environmental modeling category: IAM saw the sharpest rise (173% from 2010 to 2022), followed by water modeling (35%) and metrology and weather modeling (20%).

To examine the similarities in the use of nuanced and assertive terms, we count the percentage of shared boosters and hedgers in knowledge claims across fields, focusing on the ten most used terms per discipline. The choice of specific words to strengthen or nuance propositions has discriminatory power to distinguish between the human fields and the physical and socio-environmental modeling fields (Figures 1D and 1E). Specific boosting terms for the human (physical) sciences are “idea,” “understanding,” “conclude,” or “important” (“solution,” “predict,” “efficiency,” or “accurate”), hinting at the predominant reflective and quantitative nature of the human and physical sciences, respectively. As for hedging terms, they are “argue,” “interpret,” “view,” or “belief” (“propose,” “estimate,” “theoretical,” or “uncertain”). The most similar fields in their selection of specific hedgers and boosters are electromagnetism and hydrodynamics and water and weather modeling, respectively, sharing eight out of the 10 most frequently used terms.

### Water modeling supports claims with many more numbers than physics does

Confidence and certainty in knowledge claims are often reinforced with numerical data. Numbers impart certainty, neutrality, and epistemic authority,^21^ and their purported accuracy can be used to deflect contestation.^22^ What can be quantified can be controlled, manipulated, and tamed.^23^^,^^24^ If the strength of knowledge claims does reflect the capacity of a given field to latch onto state of affairs, we would expect the booster-to-hedger ratio and the degree of numerification of a given field to be correlated.

**Figure 2:**
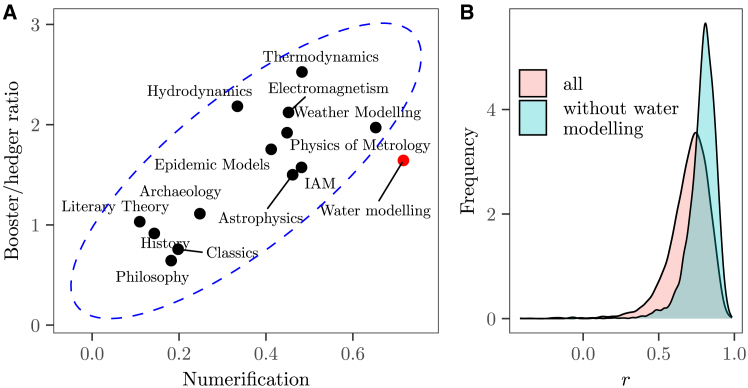
Connection between numerification and strength of knowledge claims (A) Scatterplot. The x axis shows the mean number of numbers across the six number categories considered (see text). The elliptic envelope shows the 95% confidence intervals based on Mahalanobis distances. The red dot is the outlier. (B) Density plot showing the bootstrapped Pearson correlation coefficient (*r*) after including and excluding water modeling from the calculations.

To explore this hypothesis, we tally the occurrence of numbers in knowledge claims across six categories—integers, decimals, percentages, number of decimal digits (scale), ordinals, and word numbers (excluding numbers not resulting from quantification; e.g., dates, years, centuries, and BC/AD mentions) (see algorithm in Puy et al.^25^). We observe that a stronger use of boosters is associated with higher numerification $(r_{boot}=0.7$, 95% confidence interval [CI] [0.4,0.9], $10^{3}$ resamples), with the human fields using as expected fewer numbers than the physical fields (Figure 2A). Interestingly, integrated assessment, weather, and water modeling support their claims with more numbers than even the most precise physical sciences, like thermodynamics, metrology, or electromagnetism. In fact, the combination of numerification and booster-to-hedger ratio places water modeling significantly farther from the center of the data distribution, flagging it as an outlier according to Mahalanobis distance. If we exclude water modeling from the analysis, the correlation between numerification and booster-to-hedger ratio increases $(r_{boot}=0.8$, 95% CI [0.5,0.9]) (Figure 2B).

**Figure 3:**
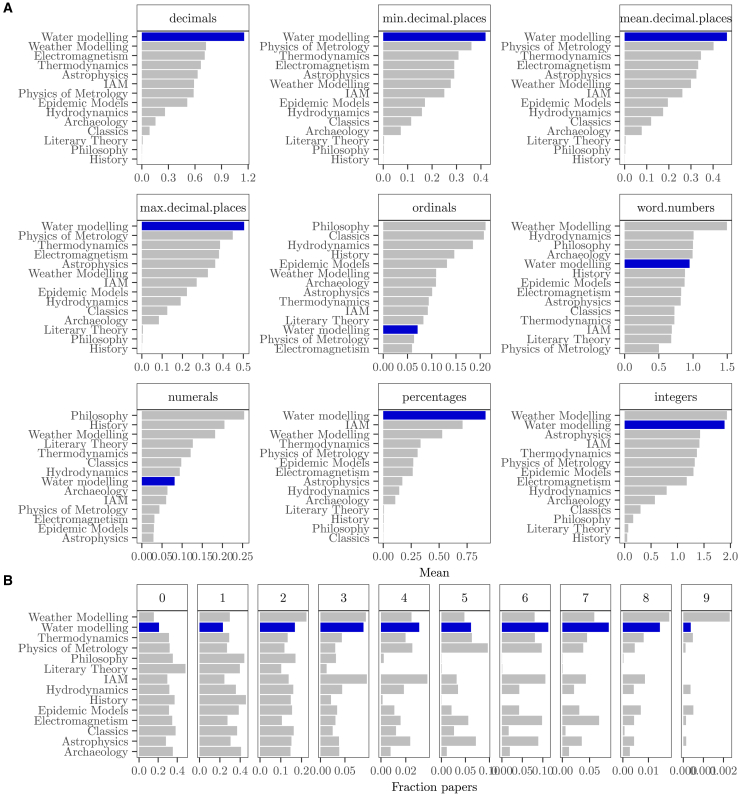
Numerification (A) Average number of decimals, ordinals, etc. per field. Note that three facets focus on the same category and show the minimum, mean, and maximum value of the number of decimal digits. (B) Fraction of papers with knowledge claims that do not include any number (0), with numbers in one category (e.g., integers), in two categories (e.g., decimals and ordinals), and up to the nine categories considered.

Water modeling ranks highest in two of the six analyzed number categories: it is the discipline whose claims rely the most on percentages and decimals (in terms of minimum, mean, and maximum number of digits) and the second discipline using more integers after weather modeling (Figure 3A). Ninety-five percent of the papers analyzed in water modeling present knowledge claims supported by up to six decimal numbers (up to four in thermodynamics and electromagnetism), up to five mentions of percentages (up to three in weather modeling), and up to eight integers (up to eight and seven in weather modeling and thermodynamics, respectively) and a mean of up to two digits after the decimal point (same for weather modeling, thermodynamics, and metrology). Water modeling also leads the proportion of claims supported by numbers in six and seven number categories simultaneously and ranks second in the proportion of claims backed up by numbers in four and eight number categories simultaneously, after integrated assessment and weather modeling (Figure 3B).

### The numbers reported in water modeling lack uncertainty and SA

Does the significantly stronger emphasis on supporting knowledge claims with numbers in these socio-environmental modeling fields imply that they quantify their object of study better than physics? Model-based numbers can indeed be very precise and reliable: those of weather modeling, for instance, are quite accurate because weather systems can be treated as physically closed systems for short periods of time, for which numerical uncertainties can be effectively measured.^26^^,^^27^ Water and integrated assessment modeling, respectively, seem to support their knowledge claims with more numbers than weather modeling and all the other physics-based disciplines. Is this numericization warranted, and are their produced numbers robust against uncertainty?

We explore this question by zooming in into water modeling, the outlier in Figure 2A and hence the field most deserving of a deeper analysis, and examining whether their numerical inferences are supported by an uncertainty and sensitivity analysis (UA/SA) in the main text.^28^ The numbers produced with a proficient UA/SA result from a comprehensive examination of the uncertainty space and an understanding of the most relevant factors responsible for the output uncertainty. Models that have undergone a stringent UA/SA are more defensible against criticisms of instrumental use for policy-based evidence, unwarranted accuracy, or overquantification,^29^ issues that resonate in the broader context of the so-called reproducibility crisis.^30^^,^^31^

**Figure 4:**
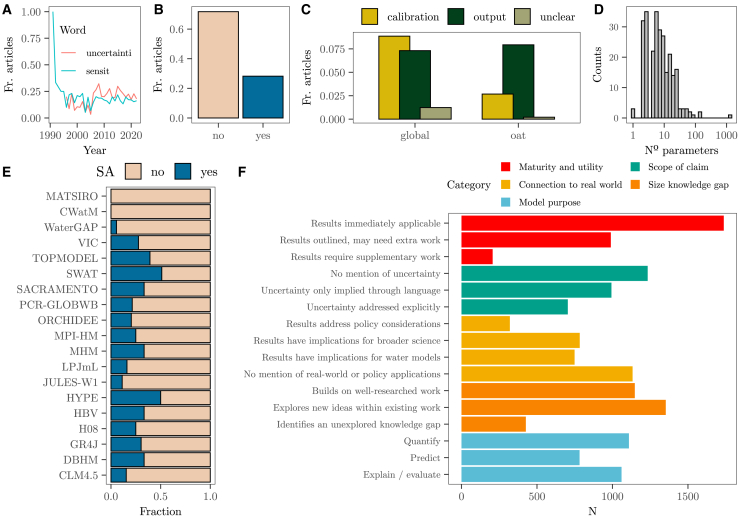
Sensitivity analysis practices in water modeling (A) Fraction of articles with the roots “uncertainti” and/or “sensit” in the abstract through time over the total number of water modeling studies analyzed (2,942). (B) Fraction of articles that conduct a technical SA over the number of papers that do include the stem “uncertainti” and/or “sensit” in the abstract (978). (C) Fraction of articles that conduct a global/OAT SA to calibrate the model or on the model output. The “unclear” label in the legend refers to studies for which we were unable to identify what the SA was conducted on. (D) Distribution of the number of parameters submitted to a technical SA on a logarithmic scale. (E) Fraction of articles that conduct a technical SA per water model. (F) Framing of uncertainties in water modeling. See the Method details and the supplementary materials for a detailed explanation of each category.

We find that only 978 papers using water models out of a total of 2,942 (∼33%) include the terms “uncertainty” and/or “sensitivity” and/or their derived words in the abstract, title, or keywords (stem being “uncertainti” and “sensit” according to Porter’s algorithm^32^) (Figures 4A and 4B). This result suggests that two-thirds of water modeling papers did not have the exploration of model uncertainties and sensitivities as a key goal. After close-reading the full text of those 978 papers, we find that only 30% conducted a technical SA in the main text. One hundred and twelve studies ran it for calibration and validation purposes (to determine which parameters an error function is most sensitive to), whereas 142 implemented it on the model output (to determine which parameters convey the most uncertainty to the model output) (Figure 4C). Notably, for calibration, water modelers lean more toward global sensitivity methods, whereas the assessment of output uncertainties is mostly done with one-at-a-time (OAT) approaches. OAT methods are discouraged by SA practitioners because they are incapable of thoroughly exploring the uncertainty space and cannot detect interactions between uncertain inputs.^33^

Most UA/SA exercises (75%) are done on 13 or fewer uncertain model inputs, with the mode and the median being three and seven uncertain inputs, respectively (Figure 4D). Given the high dimensionality of water models, these results suggest that their uncertainty space is basically left unexplored. For instance, the fraction of the uncertainty space explored with an OAT conducted on the water models PCR-GLOBWB or WaterGAP (which respectively have 44 and 30 parameters^34^^,^^35^) is indistinguishable from zero ($4.3\times 10^{-24}$ and $2\times 10^{-14}$, respectively). The same can be said if the approach is a global SA conducted on only 13 parameters out of 30, as the fraction explored is $7.6\times 10^{-6}$ (Figure S1; Table S1). Furthermore, for 75% of water models, the proportion of studies with a UA/SA is less than 30%, and some water models may have never undergone a combined UA/SA (Figure 4E). Hence about 90% of the 2,942 papers may have produced numbers without a stringent UA/SA. These results resonate with works that have attested a poor appraisal of uncertainties and sensitivities in other model-based fields, such as life cycle assessment,^36^ economy and finance, or medicine.^37^

To determine if the absence of a UA/SA is balanced by a nuanced acknowledgment of the studies’ limitations, we close-read the abstracts of the 2,942 papers that form our water modeling corpus and analyze how they frame uncertainty (Method details). Most studies present results as conclusive, rarely suggesting the need for further work, which implies confidence in model-based numbers and inferences. Quantitative uncertainties are infrequently addressed with ranges, error margins, or dispersion measures. Despite their societal relevance, a large fraction of papers do not mention any real-world or policy implication, and most only state their relevance for other water models or to other scientific fields. Overall, the emphasis on quantification and prediction outweighs the role of water models as explanatory tools (Figure 4F).

## Discussion

We present evidence that fields heavily relying on models to study complex phenomena at the intersection of the social and natural sciences, such as epidemic, integrated assessment, and water modeling, exhibit a level of assertiveness in their knowledge claims comparable to the physical sciences. Integrated assessment and water modeling also substantiate their claims with more numbers than all physical fields considered, including thermodynamics. Unless we assume that their capacity to map (and quantify) their object of study rival (and surpass) that of the most precise physical fields, we should consider that their linguistic confidence reflects an insufficient account of uncertainty and an overuse of numbers, that is, “mathiness.”^38^ Focusing on water modeling, the field most reliant on numbers to sustain its knowledge claims, we provide evidence of this deviation by attesting poor practices in uncertainty and SA and a liberal use of digits to imply precision, with little regard for model limitations. These findings align with prior evidence of false numerical accuracy in water models, particularly in irrigation modeling.^39^^,^^40^

Although our close-reading of SA practices does not extend to epidemic or IAM, some studies suggest that the accuracy of their model-based inferences may also collapse when uncertainties are properly considered (see Puy et al.^41^ and Edeling et al.^42^ for epidemic models and Saltelli et al.^43^ and Tavoni and Valente^44^ for IAMs). Our results may thus support arguments that modelers often exhibit undue confidence, which is particularly problematic when high stakes are involved, as in policy-making.^45^^,^^46^^,^^47^ The physics-like assertivity of epidemic, integrated assessment, and water modeling and the hyperbolic numeracy of the last two may reflect the “state of exception” of mathematical modeling.^48^ This condition derives from the flexibility, complexity, epistemic status, and resistance to falsification of mathematical models, which potentially creates the grounds for unchecked confidence to develop.

The “no-miracles” argument from philosophy of science (“we should be committed to our theories [models] if they are successful in the real world”^49^^,^^50^) provides another dimension to this interpretation. If we assume that propositions should align with the strength of evidence to prevent hype or understatement, then fields with a strong track record of real-world success (understood as accurate predictions and retrodictions, reliable explanations, effective interventions, and technological developments) may justify greater epistemic commitment. Although integrated, epidemic, or water modeling have shown unquestionable real-world achievements (e.g., climate change assessments,^51^ mapping of short-term infection spreading,^52^ and flood forecasting and prevention^53^), strong assertiveness and quantification in physics-based fields may be more warranted given their consistent track record and their foundational role in developing principles underpinning these achievements—including those ingrained in the modeling fields just mentioned (Method details). Only a broader and more context-specific reading of “success” (e.g., decision-making relevance, policy impact, societal influence, and contribution to public awareness) would allow to turn the “no-miracles” argument in favor of physics around. However, the assumption would then be that the success of epidemic, integrated assessment, and water modeling depends significantly on external, transient factors rather than on intrinsic properties such as the model’s validation, falsifiability, or fitness for purpose. Such reading also implies that they are more vulnerable to external influences and vested interests and hence do not dispel concerns about unwarranted confidence in their commitments and quantification.

It may also be argued that our results on confidence expressions reflect not only the nature of the domain of inquiry but also linguistic factors and the particular scientific aims of each field. Indeed, rhetoric devices are domain specific: out of the 10 most common hedgers and boosters listed for this study, the physics and the humanities only share ∼27%. While hedgers and boosters may differ among disciplines, this disparity does not necessarily extend to their role in nuancing or strengthening propositions. For instance, engineering and applied linguistics have domain-specific hype words (“crucial” and “successful,” respectively),^54^ and yet these words hype all the same. Arguing that our results reflect distinct linguistic practices (and thus that disciplines cannot be meaningfully compared) would be supported if, instead of a stratification showing an increase in booster prevalence from the humanities to physics, we observed a random arrangement of disciplines with no correlation between complexity and the treatment of doubt.^13^ However, the alignment of fields within the epistemic dimension expected for the HoS hypothesis suggests that this is not the case.

We cannot rule out the possibility that the strong assertiveness and numeracy shown by some physics fields may also reflect factors other than their high consensus and ability to map regularities. For instance, the research conducted at the frontiers of knowledge in physics is characterized by substantial levels of disagreement, theoretical exploration, and difficulty in determining which contributions will turn out to be significant.^11^ Excessive assertivity/tentativeness in this context may be used as a rhetorical device to artificially strengthen the weight of evidence in order to convince peers or hedge against criticism. However, our results are unlikely to be biased by an excessive amount of work at the frontiers of knowledge because (1) the papers in our corpus do not come from repositories but from well-established, peer-reviewed journals and (2) we have sampled physical fields with a relatively high consensus and avoided more speculative domains such as quantum mechanics or high-energy research. The same can be said for the human and the fields modeling at the intersection of disciplines that we have selected.

Our work focuses on abstracts, which may omit details found only in the full text.^55^ Due to their concise nature, abstracts often emphasize explicit claims over observations, comparisons, or nuanced statements^56^ and may neglect adverse results or potential harm.^57^ As a result, our findings may slightly overestimate the weight of boosters across fields. However, this is likely to impact the final booster-to-hedger ratio at the field level, not necessarily the pattern behind the HoS. We find it unlikely that this potential bias drives the observed trend linking stronger assertive statements with increased numerification moving from the human to the physical sciences. Furthermore, abstracts reflect the key points that the authors want to convey, thus reducing the “noise” that may be found in the results section of a given paper.

Our finding of an increase in assertivity moving from the human to the physical sciences (and hence that there may be an epistemic dimension to the HoS) should not be seen as evidence that the humanities are epistemically inferior to physics. Values, art, literature, ethics, or history are multidimensional phenomena whose complexity may be better addressed through critical inquiry, openness, reflection, and respect for plurality and therefore through tentative language in knowledge claims. Assertivity and quantification risk collapsing the breadth of human experience into artificially accurate flat statements. While strong knowledge claims in physics may be warranted due to its focus and success in highly controlled, small-world settings (a laboratory, a technological device^58^), open-ended propositions may better match the properties of open-ended realms. *A priori*, integrated, epidemic, and water modeling live in a world that shares several features with the world of the humanities: they are situated and grapple with fundamental ambiguity, human behavior, values, ethics, and societal contexts.^44^^,^^59^^,^^60^^,^^61^^,^^62^^,^^63^ It is their similar navigation between knowledge and uncertainty that has prompted calls for models to better embrace the humility inherent in the humanities.^24^^,^^26^^,^^46^^,^^64^ Although some modeling principles are physically bounded (Darcy’s law and the St. Venant equations in water modeling and energy balance equations in IAMs), the wide variety of water models^65^^,^^66^^,^^67^^,^^68^ and IAMs^69^ (from model framing and results to performance) does not seem to reflect a robust physical foundation as much as fields grappling with fundamental uncertainty.

Donella Meadows, author of the classic book “Limits to Growth,”^70^ observed 40 years ago that little seemed to have changed in modeling practices despite long-standing criticism.^71^ This inertia may no longer be tenable if scientific fields modeling at the interface of human and environmental systems are to maintain their credibility in the face of an increasingly uncertain world.

### Limitations of the study

For each physical and human field, we retrieved papers from 1990 to 2022 across ten representative journals, yielding a sample that is comprehensive but not exhaustive. Similarly, our selection of models in integrated assessment, epidemic, and water modeling represents a broad, though incomplete, overview of available models. Our list of boosters and hedgers could also be expanded with additional terms. Lastly, our study focuses on the endpoints of the HoS, omitting disciplines like chemistry or biology, which were central to the hypothesis’s original formulation. Future research could incorporate these fields to explore whether the epistemic dimension within the HoS suggested by our results holds once the assertivity of disciplines considered of medium complexity is accounted for.

## Resource availability

### Lead contact

Request for further information and resources should be directed to the lead contact, Arnald Puy (a.puy@bham.ac.uk).

### Materials availability

This study did not generate new unique reagents.

### Data and code availability

• Data: The data reported in this paper are available at Zenodo (https://zenodo.org/records/14917599).^25^ The dataset with the original Web of Science data and abstracts is subject to restrictions on its use and is governed by specific licensing agreements with the copyright holder.
• Code: The code to reproduce the results of this paper is available at Zenodo (https://zenodo.org/records/14917599).^25^
• All other requests: Any additional information required to reanalyze the data reported will be shared by the lead contact upon request.

## Acknowledgments

We thank Federico Ferretti for providing us with the astrophysics dataset. This work was funded by 10.13039/100014013UK Research and Innovation under the UK government’s Horizon Europe funding guarantee (project DAWN, PI A.P., EP/Y02463X/1).

## Author contributions

A.P. designed the manuscript and its conceptual approach, with contributions from S.N.L. and N.W. A.P. performed the bibliometric analysis, paper screening, text mining, and machine learning. A.P. produced the list of hedgers and boosters, with contributions from all other authors. A.P., D.G., and K.R.L. analyzed and tallied the frequency of hedgers and boosters in all documents and analyzed the results. E.B., S.F., E.M., and A. Sobhani designed the classification scheme to frame uncertainties, collected the data, and analyzed the results. A.P., S.L.P., R.S., A.L., and A. Saltelli designed the study of sensitivity analysis practices, collected the data, and analyzed the results. A.P. wrote the manuscript, with contributions from all other authors and from A.C., L.A.M., and A. Saltelli especially on the discussion section. All authors corrected, discussed, and revised the final version.

## Declaration of interests

The authors declare no competing interests.

## STAR★Methods

### Key resources table

**Table:** 

REAGENT or RESOURCE	SOURCE	IDENTIFIER
**Software and algorithms**		

	The code to reproduce our results can be found in Zenodo (https://zenodo.org/records/14604662https://zenodo.org/records/14604662).	

### Method details

#### Selection of scientific fields and construction of the corpora

For the human sciences, we chose philosophy, classics and literary theory due to their reflective nature and focus on open ethical, abstract and textual reasoning. History and archaeology represent a more empirical trend, particularly the latter due to its adoption of methods and techniques from earth sciences, biology and chemistry.^19^^,^^20^

For physics, astrophysics has substantiated some of our best theories with empirical evidence (e.g., general relativity through gravitational lensing and the motion of celestial bodies, confirmation of gravitational waves^72^) and produced verifiable predictions (e.g., solar eclipses, comet orbits). Electromagnetism sustains the design of household applications, telephones and antennas, making it a prominent physics-based field with real-world success confirming its accuracy. Physics of Metrology has precisely measured through experiments several constants whose value has later been used for practical purposes, such as the speed of light for optimizing high-speed data transmission systems or Planck’s constant for the development of superconducting circuits.^73^ Hydrodynamics studies the motion of liquids and has several applications in engineering, including determination of the mass flow rate of petroleum through pipelines, prediction of wave dynamics, and measurement of liquid metal flows.^74^ Thermodynamics is grounded on a solid mathematical framework to study the interactions between energy and matter and was the intellectual driving force behind the industrial revolution.^75^

With regards to the selected cross-disciplinary modeling fields, we chose epidemic, water and integrated assessment modeling because of their scientific and societal relevance and their merging of social, physical and human elements in their simulations. Weather modeling was selected as a benchmark because it is a handbook example of a physics-based modeling field with high predictive success whose outputs are widely used daily.^26^

We used the Web of Science (WoS) to retrieve the bibliographical sample for our study. For each of the fields in the human and physical sciences, we retrieved all papers published between 1990 and 2022 in ten representative disciplinary journals, which were selected based on peer advice and on the rankings provided by Scimago (Supplementary Materials). For the modeling fields, we listed the most relevant domain-specific models based on peer advice and searched for their mention in the title, abstract or keywords. We applied a different strategy in constructing the corpus for integrated assessment, epidemic and weather modeling fields compared to the other human and physical fields because 1) papers using the targeted models are spread across different journals and research areas, making it challenging to rely on a fixed list of top journals, and 2) we wanted to ensure that the modeling corpus included papers dealing with or using the targeted models, which required using the model’s name as a search query in the abstract, title or keywords. We provide the full WoS search queries in the Supplementary Materials.

Overall, the final bibliographical sample retrieved at this stage included 753,383 papers (Figure S2). Given the detailed analysis in the case of water modeling, the retrieval of the bibliographical sample required a more fine-grained approach, which we outline in the sub-section below.

#### Construction of the water modeling corpus

We compiled a comprehensive list of 19 relevant large-scale water models based on works by Addor and Melsen^76^ and Telteu et al..^66^ We excluded newly published models or models without a consolidated publication record, such as HydroPy (an updated version of MPI-HM published in 2021 by Stacke et al.^77^) and Water And ecosYstem Simulator (WAYS, published in 2019 by Mao et al.^78^).

To conduct the bibliometric analysis we performed a Web of Science search for each model, targeting studies up to the end of December 2022 that included the model name in the title, abstract or keywords. The search queries followed the format recommended by Addor and Melsen,^76^ with the model acronym or the full model name and the term “model”. In some cases, we excluded the model acronym from the query to avoid irrelevant matches, such as papers about ethnic groups in Swat, North Pakistan (for the SWAT model).

The resulting bibliographical sample consisted of 3,403 studies. Out of these, 82 papers (∼2%) were found to have duplicated references, with 71, eight, two, and one paper allocated to two, three, four and five different model-categories, respectively. To address this overlap, we randomly assigned the duplicated papers to a single model-category, resulting in a reduced sample of 3,306 papers.

To ensure that the papers used the corresponding water model, we examined the sentences preceding and following the mention of the model name in the abstract. We considered the model as being used when the text indicated model-based results, utilization of model outputs as input in another modeling exercise (e.g., soil moisture, water consumption estimates) or model comparisons involving the model under study. In cases where the model name was not found in the abstract but the paper clearly focused on water-related topics, we searched the full text for confirmation based on the mentioned criteria. This screening process helped identify and exclude studies that did not meet the specified criteria, resulting in a refined pool of 2,942 papers representing the water modeling field. After being added to the rest of studies, the final corpus amounted to 756,325 papers spanning 14 academic fields over thirty years.

#### Extraction of knowledge claims through machine learning

We employed the SciBERT model,^16^ a variant of BERT pre-trained on scientific text (1.14M papers, 3.1B tokens), to extract knowledge claims from academic abstracts. The goal was to programatically classify sentences in abstracts as either knowledge claims or non-knowledge claims. We defined knowledge claims as sentences presenting the paper’s findings, results or reflections, often phrased as “Here we show that …”, “this paper suggests that …”, “we observe that …”, “I argue that …”. Sentences situating the research within the state-of-the-art or explaining methodology were excluded from this category.

We randomly sampled 200 abstracts from each scientific field from the corpus of 755K documents and broke down the abstract into sentences using regex. Half of the sample was used to train the model and the other half was used for validation purposes. We manually labeled a sentence with a 1 if it was a knowledge claim and with a 0 if it was not, for a total of approximately 17,000 sentences. Once the labeling was completed, we utilized the BertForSequenceClassification model from the Hugging Face transformers library,^79^ initialized with SciBERT weights (allenai/scibert_scivocab_uncased). This model is well-suited for sequence classification tasks in scientific domains.

We tokenized the sentences using the SciBERT tokenizer (BertTokenizer), fine-tuned to handle the vocabulary specific to scientific literature. The tokenization settings included a maximum length of 128, with padding and truncation to ensure a uniform input size. We trained our model using a training loop with three epochs, a batch size of 16 for both training and validation and a warmup strategy with a learning rate scheduler, starting at 5e-5 and decaying linearly after the warmup period. We also used AdamW optimizer with weight decay to prevent overfitting.

We evaluated the model after each epoch on the validation dataset and calculated performance metrics such as accuracy (0.92), precision (0.93), recall, F1-score (0.88) and validation loss (0.24). These metrics indicate an excellent performance with very few false positives and false negatives.

#### Selection of hedging and boosting terms

To analyze the balance of doubt and certainty across scientific fields, we identified hedging and boosting terms commonly used in academic papers and quantified their prevalence in all the knowledge claims extracted via machine learning. Hedgers are expressions that indicate hesitancy, acknowledging the conditional or subjective nature of results and their limitations. Examples include terms like may, could, suggest, believe, possible, presume, few, believe and opinion, which intentionally convey ambiguity and humility while respecting alternative claims.^9^ In contrast, boosters demonstrate strong commitment to a claim, leaving little room for uncertainty. They emphasize specific propositions, result reliability or research significance. Examples include terms like affirm, assert, show, accurate, important, always, advancement and verification. Hedgers and boosters reflect scholars’ commitment on knowledge claims and help strike a balance between collegial attitude and explicit involvement with a specific community.^8^

We utilized ChatGPT-3.5 to compile a preliminary list of the 50 most common hedging and boosting verbs, nouns, adjectives, adverbs, quantifiers/determiners and modal verbs in academic writing.^80^ We filtered out neutral terms and those with unclear hedging or boosting effects (e.g., “analysis”). Additionally, we cross-checked and expanded the list with words from similar studies.^8^^,^^9^^,^^17^^,^^81^^,^^82^ The final list comprised 196 hedging and 176 boosting terms and can be found in the Supplementary Materials (Tables S2 and S3). To check the robustness of the classification, five authors (SLP, EB, SF, EM, ASob) independently assigned each term to either the hedging or boosting category, achieving an agreement of ∼95% with the original grouping. We then analyzed the list of hedgers and boosters against each of the 756,325 abstracts forming our corpus, tallying the frequency of each pattern occurrence.

#### Counting of numbers

To quantify the occurrence of numerical expressions in knowledge claims, we developed an R-based algorithm (counting.script.R, see Puy et al.^25^). The function first identifies and counts all numeric values containing decimal points using a regular expression pattern. A secondary check counts the number of decimal places for each decimal number, including those expressed in scientific notation. Then, ordinals (e.g., “first”), cardinal numbers (e.g., “ten”) and specific terms (e.g., “once”, “twice”) are matched and counted using predefined lists of words. A separate function identifies integers while excluding years, centuries and numeric expressions related to historical time references (e.g., “20th century”, “300 BC”) and measurements (e.g., “km”). Finally, percentages are detected using patterns that match numeric percentages (e.g., “5%”, “5%”, “per cent”). A wrapper function aggregates all counts and outputs the total occurrences of each numeric category.

#### Study of sensitivity analysis practices

To explore whether and how water modellers conduct Sensitivity Analysis (SA, the examination of which uncertain input/model structures convey the most uncertainty to the model output^28^), we retrieved studies containing the term “uncertainti” and/or “sensit” in the abstract, resulting in 978 papers (33% of the total water modeling sample). This screening strategy aimed to identify studies that conducted an SA and studies that conducted an SA but referred to it as an “uncertainty analysis”, a conflation well documented in the modeling literature.^37^ We finally kept 971 papers after removing studies whose main text was inaccessible due to language barriers (e.g., Chinese), paywalled access or because the title referred to a press release/conference abstract and not to a scientific manuscript.

Following previous works,^36^^,^^37^ we close-read the main text and categorized the 971 papers in two groups: those that did not conduct a formal SA and those that did. We defined a “formal SA” as any analysis that quantifies the uncertainty contribution of specific uncertain parameters or structures to the overall output uncertainty of a water model. Hence we excluded studies that.

(1) Only conducted an uncertainty analysis (i.e., Monte-Carlo-based works, factorial analyses, model ensemble analyses) without checking how much each of the uncertain parameters influence the model output uncertainty. That is, UA without SA.
(2) Examined output sensitivity to a single uncertain parameter/structure [i.e., to different global climate models, Digital Elevation Models (DEMs), effective soil depths]. Such approach does not allow to establish a ranking of parameters in terms of their effect to the output uncertainty, a key goal of SA.
(3) Used a water model output (i.e., the extent of tropical floodplains) as a specific input in their modeling exercise and then conducted the SA on the resulting model output.

For studies that fulfilled our definition of “formal SA”, we specified whether the SA was “One-At-a-Time” (OAT, when the uncertain parameter/structure is varied along their uncertainty range while all the other uncertain parameters/structures are kept fixed), or “global” (when all uncertain parameters/structures are varied at once). Global SA approaches do a better job than OAT methods in exploring the model’s uncertainty space because they are able to appraise interactions, which are missed by OAT approaches. The latter are only reliable when the model is additive (when the output variance can be decomposed as the sum of individual effects).^33^ This condition is rarely met in environmental/water models given that multiplications and exponents are enough to cause non-additive behavior.

If the SA approach was unclear due to insufficient explanation or a bespoke methodology, we classified it as “global”. We also specified whether the SA was conducted on the model output or on a given error function for calibration purposes (i.e., Nash-Sutcliffe efficiency criteria) and the number of explored uncertain parameters/structures.

#### Framing of uncertainty

To cross-check the results obtained with the counting of hedgers, boosters and numbers, we analyzed the confidence exhibited by water modellers in their knowledge claims by adapting the uncertainty frames by Guillaume et al..^83^ We assessed the belief of water modellers in 1) the maturity and utility of their knowledge claims (“to what extent can we use the results?”), 2) the scope of their claims (“how much confidence do we have in our results?”), 3) the connection of their claims with the real-world (“can results guide policy-making?”), 4) the size of the knowledge gap addressed by the paper (“do we know anything about the topic?”), and 5) the purpose of the model (“why do we use the model for?”). We divided each of these sections into sub-categories covering a continuum from low to high confidence (e.g., a, b, c), and conducted the analysis at the sentence level. A document could therefore be classified under categories 1a, 2a, 3b, etc, but could not be assigned to both 1a and 1b, or 3a and 3c simultaneously.

We present the final classification scheme and the explanation of the criteria used to categorize papers in each sub-category in the Supplementary Materials. The classification scheme resulted from an iterative process in which four authors (EB, EM, SF, ASob) randomly close-read 30 abstracts. They classified each paper into its different sub-category, validated the results through group discussions and fine-tuned the taxonomy. This process was repeated three times until an overall agreement of over 75% was achieved (where at least three out of four authors agreed on the allocation of a given paper to a specific sub-category). After this robustness check, the abstracts of all 2,942 papers were read and each paper was classified according to the final classification scheme.

### Quantification and statistical analysis

There are no statistical analysis or quantification to include in this study.
