# Supplementary material for: Socio-environmental modeling shows physics-like confidence with water modeling surpassing it in numerical claims
Source: iScience. 2025 Mar 13;28(4):112184. doi: 10.1016/j.isci.2025.112184 (PMC11986976; doi:10.1016/j.isci.2025.112184)
Supplement: Document S1. Figures S1 and S2, Tables S1–S3, and Data S1 [file mmc1.pdf]

iScience, Volume *28*

Supplemental information

Socio-environmental modeling shows physics-like confidence with water modeling surpassing it in numerical claims

Arnald Puy, Ethan Bacon, Alba Carmona, Samuel Flinders, David Gefen, Mohammad Khanjani, Kai R. Larsen, Alessio Lachi, Seth N. Linga, Samuele Lo Piano, Lieke A. Melsen, Emily Murray, Razi Sheikholeslami, Ariana Sobhani, Nanxin Wei, and Andrea Saltelli

Figures

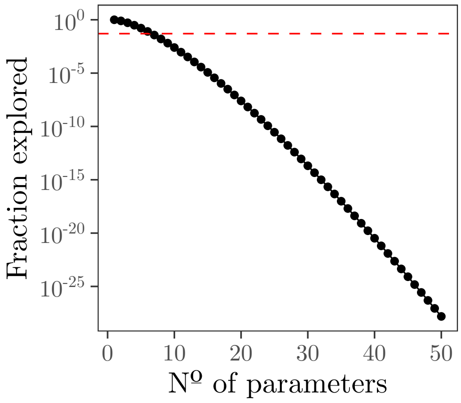

Figure S1: Fraction of the uncertainty space explored with an OAT as a function of the model dimensionality. The red, dashed horizontal line is at 0.05 (5%).

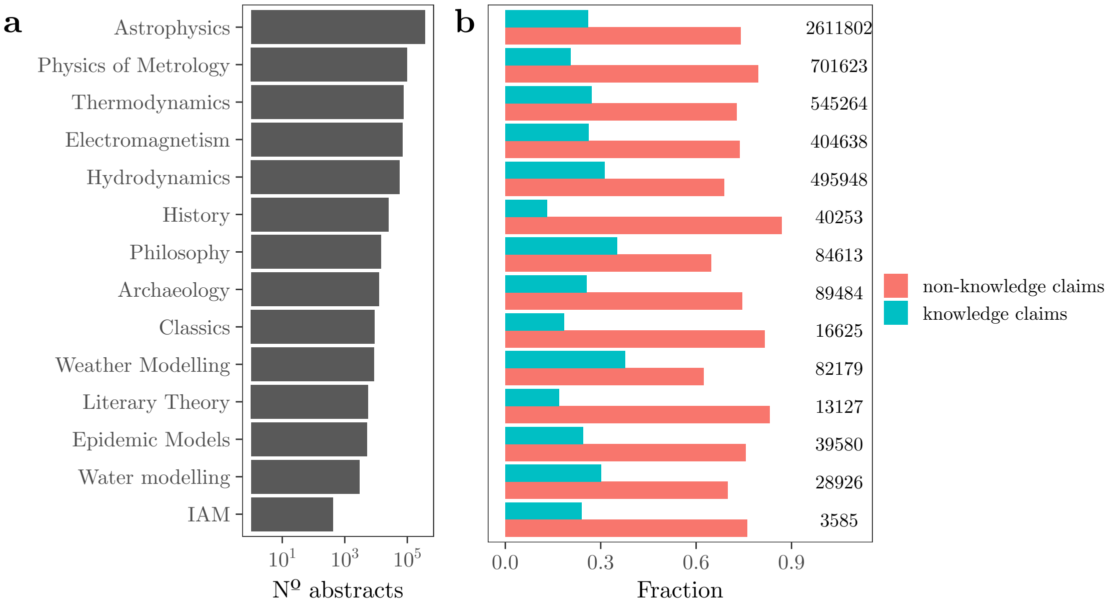

Figure S2: Descriptive statistics. a) Total number of abstracts per field. The x-axis is in $\log_{10}$ base. b) Bar plot showing the fraction of sentences presenting knowledge claims versus those that do not, per field. The total number of sentences examined is displayed on the right.

Tables

Table S1: Fraction of the uncertainty space explored with an OAT as a function of the number of uncertain parameters k.

k	Fraction
5	1.64×10^{-1}
10	2.49×10^{-3}
15	1.16×10^{-5}
20	2.46×10^{-8}
25	2.85×10^{-11}
30	2.04×10^{-14}
35	9.74×10^{-18}
40	3.28×10^{-21}
45	8.13×10^{-25}
50	1.54×10^{-28}

Table S2: List of hedgers.

ID	Hedger
1	a few
2	a good deal of
3	a great deal of
4	a large amount of
5	a large number of
6	a limited number of
7	a majority of
8	a number of
9	a range of
10	a significant number of
11	a small number of
12	a variety of
13	acknowledge
14	alleged
15	almost all
16	almost every
17	an array of
18	an extensive number of
19	an impressive number of
20	apparent
21	apparently
22	approximation
23	arguable
24	arguably
25	argue
26	assumably
27	assume
28	assumption
29	belief
30	believably
31	believe
32	certain
33	certain types of
34	conceivable
35	conceivably
36	conception
37	conceptual
38	conditional
39	conjectural
40	conjecturally
41	conjecture
42	consider
43	considerable
44	consideration
45	contemplate
46	contention
47	controversial
48	could
49	countless
50	debatably
51	debated
52	differing levels of
53	doubt
54	doubtful
55	doubtfully
56	enough
57	equivocal
58	estimable
59	estimate
60	estimation
61	expectation
62	explore
63	feasible
64	few
65	frequently
66	hypothesis
67	hypothesize
68	hypothetical
69	hypothetically
70	implied

71	imply
72	impression
73	improbable
74	improbably
75	in some cases
76	inclination
77	indirectly
78	inexact
79	infer
80	inference
81	inferred
82	inferredly
83	insinuate
84	interpret
85	large
86	likelihood
87	likely
88	many
89	many different types of
90	many kinds of
91	many types of
92	may
93	maybe
94	might
95	most
96	most types of
97	nearly all
98	note
99	numerous
100	often
101	opine
102	opinion
103	ought to
104	partial
105	perceived
106	perceptibly
107	perception
108	plausible
109	plenty of
110	ponder
111	possibility
112	possible
113	possibly
114	postulate
115	postulation
116	potentially
117	preliminary
118	pressupose
119	presumably
120	presume
121	presumed
122	presumption
123	probabilistic
124	probabilistically
125	probability
126	probable
127	proposal
128	propose
129	proposition
130	purported
131	putative
132	putatively
133	puzzle
134	questionable
135	quite a few
136	rare
137	rationale
138	reflect
139	reportedly
140	seem
141	seemingly
142	several
143	shall

144	should
145	some
146	some kinds of
147	some types of
148	speculate
149	subjective
150	suggest
151	suggestedly
152	suggestion
153	suppose
154	supposedly
155	supposition
156	surmise
157	suspect
158	suspicion
159	tangibly
160	tentative
161	tentatively
162	tentativeness
163	the majority of
164	the most
165	the vast majority of
166	theoretical
167	theoretically
168	theorize
169	theory
170	think
171	thought
172	uncertain
173	uncertainty
174	uncorroborated
175	uncorroboratedly
176	undecidedly
177	understand
178	undetermined
179	unexpected
180	unlikely
181	unproven
182	unresolved
183	unsure
184	unsurely
185	vague
186	vaguely
187	vagueness
188	variability
189	variable
190	variation
191	various
192	variously
193	vast
194	view
195	virtually
196	would

Table S3: List of boosters.

ID	Booster
1	accentuate
2	accurate
3	actually
4	advancement
5	advantage
6	affirm
7	always
8	amplify
9	anticipate
10	assert
11	augment
12	authoritative
13	benefit
14	calculate
15	calibration
16	certainly
17	challenge
18	clarify
19	clear
20	clearly
21	compelling
22	comprehensive
23	conclude
24	conclusively
25	confirm
26	considerably
27	consolidate
28	contend
29	contradict
30	conviction
31	convincing
32	corroborate
33	creativity
34	crucial
35	decidedly
36	defend
37	definite
38	definitely
39	definitive
40	demonstrate
41	depict
42	determine
43	development
44	discovery
45	document
46	doubtless
47	effectively
48	effectiveness
49	efficiency
50	elegant
51	elucidate
52	emphasize
53	empirical
54	endorse
55	enforce
56	engaging
57	enhancement
58	enlighten
59	entirely
60	essential
61	essentially
62	establish
63	evidence
64	evident
65	exceptional
66	exceptionally
67	explain
68	extensively
69	extraordinary
70	find

71	foster
72	fundamentally
73	generally
74	highlight
75	highly
76	idea
77	illustrate
78	impact
79	important
80	impressive
81	improvement
82	incontestable
83	increase
84	increasingly
85	indeed
86	indicate
87	informative
88	innovation
89	innovative
90	innovatively
91	insight
92	insightful
93	inspiring
94	instructive
95	interesting
96	justify
97	know
98	maintain
99	manifest
100	motivate
101	notable
102	notably
103	noteworthy
104	noticeably
105	novel
106	novelty
107	obvious
108	obviously
109	outstanding
110	overwhelmingly
111	particularly
112	performance
113	persuasive
114	pioneering
115	potential
116	powerful
117	predict
118	profound
119	progress
120	prominently
121	promising
122	promote
123	proof
124	prove
125	quality
126	reaffirm
127	really
128	rebut
129	recommend
130	recommendation
131	refute
132	reinforce
133	relevant
134	remark
135	remarkable
136	remarkably
137	reveal
138	rigorous
139	robust
140	show
141	significant
142	significantly
143	solution

144	sophisticated
145	stimulating
146	strategy
147	stress
148	strikingly
149	strongly
150	substantial
151	substantially
152	substantiate
153	success
154	successfully
155	support
156	surely
157	sustainability
158	thought-provoking
159	tremendously
160	undeniable
161	undeniably
162	underscore
163	understanding
164	unequivocally
165	unquestionably
166	urgently
167	useful
168	validate
169	validation
170	valuable
171	verification
172	verify
173	very
174	vital
175	well-researched
176	will

Data S1/Additional text

DATA S1/Methods S1: OAT does not work: the geometric proof (Fig. 4)

The proof that One-At-a-Time (OAT) sensitivity analysis is incapable to systematically explore the uncertainty space is given by Saltelli and Annoni [1]. To summarize, in a high-dimensional model whose uncertainty space is geometrically represented by an hypercube, an OAT can only explore the fraction of an hypersphere in the hypercube because its sampling points are internal to the sphere by design. The volume of the hypersphere of radius 1/2 in k dimensions is given by

$$r(k) = \frac{\pi^{\frac{k}{2}}}{\Gamma\left(\frac{k}{2}+1\right)}\left(\frac{1}{2}\right)^k \tag{1}$$

Hence the fraction of the uncertainty space explored by an OAT plummets with the addition of every extra dimension. In two dimensions, the fraction explored by OAT is 0.78; in five dimensions, 0.16; in ten dimensions, 0.002. Although this is already indistinguishable from zero for all practical purposes, we calculated the fraction of the uncertainty space explored by an OAT for a model with 44 uncertain parameters such as PCR-GLOBWB [2] (4.3×10^{-24}) to illustrate the extent of the problem (Fig. S1, Table S1). See also Puy and Saltelli [3].

DATA S1/METHODS S2: Framing of uncertainty: classification scheme (Fig. 4)

Here we provide a more in-depth explanation of our framing of uncertainty analysis. Each abstract was classified in one of the sub-categories for maturity and utility of knowledge claims, scope of claims, connection of claims with real-world, size of knowledge gap addressed by paper and purpose of model based on the information contained in the abstract. The sentences used in the examples below serve to illustrate our classification rationale.

1. Maturity and Utility: To what extent can we use the results?

   (a) Results are immediately applicable with no additional efforts required.

   (b) Results are outlined, but implementation may necessitate additional work.

   (c) Results require supplementary work to substantiate the knowledge claim.

   For an abstract to be placed into (a), there should be no mention of further work needed for the results to be applicable, this also includes when the results are flatly stated. To place an abstract into (b) or (c), there should be tentative language such as mentions of assumptions, biases, parameter-dependency, limits for applications (spatial, temporal, limits regarding size of data the model is suitable for), etc. Abstracts on the extreme end of the point above would fall into (c), where there must be stress placed on the limits for application and the need for better understanding.

   Examples:
   1a: WOS000841966900005: *It is found that after bias correction, the amount, frequency, intensity and variance of the precipitation from the regional climate model resemble the observation better.* = Results have been improved and are ready to use.

   1b: WOS000284974600019: *Results indicate that the swat model could simulate streamflow at both scales reasonably well with very little difference between the observed and computed values. However, the results also indicate that there may be greater uncertainty in swat streamflow estimates as the size of the watershed increases.* = Tentative language surrounding the applicability of the results presents the limitations of the results but there is still no explicit call for supplementary work.

   1c: WOS000495598400073: *Further studies focus on more diversified model structures and watersheds conditions are necessary to verify the superiority of applying time-varying parameters.* = This request for more research in the approach of involving time varying parameters proposed in this abstract demonstrates the lack of maturity in its results

2. Scope of claim: how much confidence do we have in our results?

   (a) No mention of uncertainty.
   (b) Uncertainty is only implied through language.
   (c) Uncertainty is addressed explicitly.

   For an abstract to be categorised into (a) it cannot have any mention, explicit or implicit, of uncertainty. This means that it should not include the word "uncertainty" with explicit reference to the results, numerical ranges (including < or > forms), measures of dispersion (confidence intervals, standard deviations), etc. To qualify for (b), a paper should not have any explicit mention of uncertainties like in (a), but might include language with hedging terms (e.g., may, could, might, suggests, implies) or qualifying statements (e.g., "it appears that", "it is possible that", "this would lead us to believe...", "which mostly addresses", "in general"...). We did not included the entire list of hedgers as detailed from page 15 of the SM since this was covered in another section of the paper. To be classified into (c), the paper should have an explicit reference to uncertainties, either quantitative or qualitative.

   Examples:
   2a: WOS000348460000012: *Results showed significant intermodel differences in snowmelt efficiency and sublimation efficiency, and models with high rate of snow accumulation and melt were able to reproduce the observed seasonal evolution of SWE.* = No mention of uncertainty.

   2b: WOS000494681900002: *An evaluation of appropriate land surface spin-up period in order to investigate the optimum stabilization period for clm4.5, indicates a possible weak coupling between land and atmosphere components in model simulation.* = "a possible weak coupling" indicates uncertainty.

   2c: WOS000278308100014: *Agreement between climate projections (n=9), not accounting for deforestation and fire, in 2050 and 2098 was relatively low for the directional change in basin-wide nbp (19-37%) and aboveground live biomass (13-24%). the largest uncertainty resulted from climate projections, followed by implementation of ecosystem dynamics and deforestation* = Use of ranges within the abstract is an explicit showcase of its uncertainty.

3. Connection to the real world: to what extent can results inform policy-making?

   (a) Results explicitly address and inform policy considerations.
   (b) Results are framed as having implications for broader scientific disciplines (e.g., biology, engineering).
   (c) Results are specifically applicable to water mode
   (d) No mention of any direct connection or applications of results in the real-world or policy-making contexts.

   To be placed into (a), an abstract must present present direct statements related to policy-making or metrics introduced by governments or international organisations. Abstracts should use terms indicating applicability in decision-making, such as "policy implications", "policy recommendations" or "decision support". To be placed into (b), abstracts should identify trends or analyses that extend beyond the scope of water modelling, or explicitly mention broader scientific disciplines either by naming them or indicating general scientific applicability. They might also highlight information that apply to fields that do not require a water model. To be categorized into (c), abstracts must have a direct mention or recommendation for water modelling, such as for hydrological forecasting or validation. They must explicitly make statements linking the results to water modelling through sentences such as "applicable to hydrological studies", "relevant to water modellers", etc. To fit into (d), abstracts should have no mention of explicit statements or recommendations for practical applications, or lack indications that the results have implications beyond their own context.

   Examples

3a: WOS000735093800001: *The results from this study could be useful for policymakers and river basin authorities for the optimum planning and management of water resources under a changing climate.*

3b: WOS000384419400002: *This work opens new perspectives for better predictions of the land carbon budgets.*

3c: WOS000603315900001: *The presented procedure can be applied to any hydrological model.*

3d: WOS000457439200013: *In the hydrograph recession, new model presented* $r^2 = 0.75$*, against 0.52 obtained in its previous version.* = This was the last sentence of the abstract which is usually where there will be connections and here there is none.

4. Size of knowledge gap.

   (a) Knowledge gap is presented as well explored, building off well-researched work in that area.
   (b) Work has been done in this area, but the author is exploring new ideas/perspectives.
   (c) Knowledge gap is presented as unexplored with very little or no information available.

An abstract was allocated to (a) if the research used existing water models in intended ways, for example, comparing existing models to each other or real-world data, or predicting scenarios within a model's remit. For an abstract to be allocated to (b), the research needs to rework models outside their normal remit. For example, implementing models outside their usual function/purpose, adding to models (e.g. "this model has a simple interpretation for evaporation, we have supplemented the model with a more involved interpretation for evaporation"), or combining models or methodology to get a more in-depth prediction. If abstracts introduced completely new models, the issue was framed as currently unsolved, or understanding of scenarios was presented as very poor, then the abstract was allocated to sub-category (c). If an issue was framed as currently unsolved or understanding of scenarios is poor, it would only be considered for category (c), regardless of whether existing models are used or reworked.

Examples

4a: WOS000598690400001: *We applied the 'p model'-a parameter-sparse and extensively tested light use efficiency (lue) model, driven by co2, climate and remotely sensed greenness data-at 29 sites with multi-year eddy-covariance flux measurements.* = uses the well-known p-model within its standard capabilities.

4b: WOS000376934900001: *We explore coupling to a configurable subsurface reactive transport code as a flexible and extensible approach to biogeochemistry in land surface models. a reaction network with the community land model carbon-nitrogen (clm-cn) decomposition, nitrification, denitrification, and plant uptake is used as an example. we implement the reactions in the open-source pflotran (massively parallel subsurface flow and reactive transport) code and couple it with the clm.* = reworks model outside normal remit.

4c: WOS000451531700127: *Properly adjusting the irrigated crop area pattern to optimally use the limited amount of water available. to that avail, a sequential hydro-economic model has been developed and applied to the agriculturally intensively used zarrine river basin (zrb), iran. in the first step, the surface and groundwater resources, especially, the inflow to the boukan dam, as well as the potential crop yields are simulated using the soil water assessment tool (swat) hydrological model, driven by gcm/qm-downscaled climate predictions for three future 21th-century periods under three climate rcps* = a novel approach.

5. Purpose of model.

   (a) Quantify.
   (b) Predict.

(c) Explain / Evaluate.

If there is no mention to forecast but the abstract discusses numbers or model-based figures (it quantifies something), the paper was allocated to (a). If the abstract explicitly mentioned that the model are predicting something in the future, the paper was allocated to (b). If none of the beforementioned applied, the paper was allocated to (c) Example scenarios when a paper was allocated to (c) include statements like "this parameter conditions the other", "this explains the following", etc. In cases where the abstract both quantifies and predicts (as was naturally the case in many abstracts), it was assigned to (b) only.

Examples

5a: WOS000683143000001: *The evaluation of the swat model presented that the annual surface runoff increased by 43.53 mm, groundwater flow declined by 27.58 mm, and lateral flow declined by 5.63 mm.*

5b: WOS000441449100001: *We found that discharge will increase due to increases in precipitation between the past (1986-1995) and future (2022-2031).* = Future events are being quantified.

5c: WOS000281758700002: *An important question is how well vic runoff simulations serve to answer questions about hydrologic changes in smaller streams, which are important habitat for many fish species.* = Purpose of the model is to explore hydrological questions.

Journals selected

Philosophy

1. American Philosophical Quarterly
2. Australasian Journal of Philosophy
3. Mind
4. Canadian Journal of Philosophy
5. Erkenntnis
6. Inquiry: An Interdisciplinary Journal of Philosophy
7. Philosophical Review
8. Philosophical Quarterly
9. Philosophical Studies
10. Synthese

Classics

1. Journal of Roman Studies
2. Classical World
3. Journal of Hellenic Studies
4. Mnemosyne
5. Classical Quarterly
6. Classical Journal
7. Classical Philology
8. Greek Roman and Byzantine Studies
9. Classical Bulletin
10. Greece Rome

History

1. American Historical Review
2. Journal of Modern History
3. Journal of American History
4. Historical Journal
5. History and Theory
6. Social History
7. Speculum: a Journal of Medieval Studies
8. Journal of Global History
9. Comparative Studies in Society and History

Literary Theory & Criticism

1. Literary Theory & Criticism
2. Philosophy & Literature
3. Mississippi Quarterly
4. Criticism: A Quarterly for Literature and The Arts
5. Cambridge Quarterly
6. Clio: A Journal of Literature History and the Philosophy of History
7. Literature and Theology
8. Literator: Journal of Literary Criticism, Comparative Linguistics and Literary Studies
9. Papers on Language and Literature
10. Routledge Interdisciplinary: Perspectives on Literature

Archaeology

1. Archaeological and Anthropological Sciences
2. Archaeometry
3. European Journal of Archaeology
4. Journal of Archaeological Method and Theory
5. Journal of Archaeological Science

6. Journal of Archaeological Research
7. Journal of Field Archaeology
8. Journal of World Prehistory
9. Environmental Archaeology
10. World Archaeology

Astrophysics

1. Monthly Notices of the Royal Astronomical Society
2. Physical Review Letters
3. Astronomy and Astrophysics Review
4. Astrophysical Journal Letters
5. Physical Review D
6. Nature Astronomy
7. Astrophysical Journal
8. Journal of Cosmology and Astroparticle Physics
9. Publications of the Astronomical Society of the Pacific
10. Journal of High Energy Astrophysics

Physics Metrology

1. IEEE Transactions of Instrumentation and Measurement
2. International Journal of Termophysics
3. Journal of Instrumentation
4. Journal of Research of the National Institute of Standards and Technology
5. Measurement
6. Measurement Science Review
7. Metrologia
8. Measurement Science and Technology
9. Review of Scientific Instruments
10. Sensors and Actuators A Physical

Hydrodynamics

1. Journal of Fluid Mechanics
2. Physics of Fluids
3. Journal of Hydrodynamics
4. Journal of Hydraulic Research
5. Computers Fluids
6. International Journal of Multiphase Flow
7. Journal of Hydraulic Engineering
8. Physics of Fluids
9. International Journal of Fluid Mechanics Research
10. Experimental Thermal and Fluid Science

Electromagnetism

1. IEEE Transactions on Electromagnetic Compatibility
2. Journal of Electromagnetic Waves and Applications
3. IEEE Transactions on Magnetics
4. IEEE Transactions on Antennas and Propagation
5. Electromagnetics
6. Progress in Electromagnetics Research pier
7. International Journal of Antennas And Propagation
8. Electromagnetic Biology and Medicine
9. IEEE Journal of Electromagnetics RF and Microwaves in Medicine and Biology
10. IEEE Antennas and Wireless Propagation Letters

Thermodynamics

1. Journal of Chemical Thermodynamics
2. Thermochimica acta
3. International Journal of Thermodynamics
4. Entropy
5. Journal of Thermal Analysis and Calorimetry
6. Fluid Phase Equilibria
7. Heat Transfer Research
8. Chemical Engineering Science
9. Journal of Non Equilibrium Thermodynamics
10. Thermodynamics

For the modelling fields, we retrieved relevant documents using the following search queries:

Weather modelling

- *WC="Meteorology & Atmospheric Sciences" AND (TS="Global Forecast System" OR TS="European Centre for Medium-Range Weather Forecasts" OR TS="North American Mesoscale Model" OR TS = "High-Resolution Rapid Refresh" OR TS = "Weather Research and Forecasting" OR TS = "Unified Model" OR TS = "Global Environmental Multiscale Model" OR TS = "ICOsahedral Nonhydrostatic model" OR TS = "Action de Recherche Petite Echelle Grande Echelle") AND TS = weather*

Integrated Assessment Modelling

- *TS = (("WITCH" AND "Integrated Assessment Model*") OR "World Induced Technical Change Hybrid model" OR ("FUND" AND "Integrated Assessment Model*") OR "Framework for Uncertainty, Negotiation and Distribution" OR ("DICE" AND "Integrated Assessment Model*") OR "Dynamic Integrated Climate-Economy" OR ("MAGIC" OR "Model for the Assessment of Greenhouse Gas Induced Climate Change") AND "Integrated Assessment" OR ("POLES" AND "Integrated Assessment Model") OR "Prospective Outlook on Long-term Energy Systems" OR ("GLOBIOM" AND "Integrated Assessment Model*") OR "Global Biosphere Management Model" OR ("GCAM" OR "Global Change Assessment Model") AND "Integrated" OR "Asia Pacific Integrated Model" OR ("IMAGE" OR "Integrated Model to Assess the Global Environment") AND "Integrated Assessment Model" OR ("MAgPIE" AND "Integrated Assessment Model*") OR "Model of the Anthropogenic Global Process of Interaction of the Environment" OR ("IMACLIM" AND "Integrated Assessment Model*") OR "Integrated Model to Assess the French Low Carbon Strategy" OR "TIMES Integrated Assessment Model" OR ("MESSAGE" OR "Model for Energy Supply Strategy Alternatives and their General Environmental Impact") AND "Integrated Assessment Model") AND PY = (2002-2022)*

Epidemiological Modelling

- *TS = (("SIR* model*" OR "SEI* model*" OR "SIS model*") AND (epidemi* OR virus OR disease)) AND PY = (2000-2022)*

References

[1] A. Saltelli and P. Annoni. “How to avoid a perfunctory sensitivity analysis”. *Environmental Modelling and Software* 25.12 (2010), 1508–1517. DOI: 10.1016/j.envsoft.2010.04.012.

[2] F. C. Sperna Weiland, J. A. Vrugt, R. L. van Beek, A. H. Weerts, and M. F. Bierkens. “Significant uncertainty in global scale hydrological modeling from precipitation data errors”. *Journal of Hydrology* 529 (2015), 1095–1115. DOI: 10.1016/j.jhydrol.2015.08.061.

[3] A. Puy and A. Saltelli. “Mind the Hubris: Complexity Can Misfire”. *The Politics of Modelling. Numbers Between Science and Policy*. Ed. by A. Saltelli and M. D. Fiore. Oxford: Oxford University Press, 2023, 51–68. DOI: 10.1093/oso/9780198872412.003.0004.
